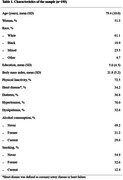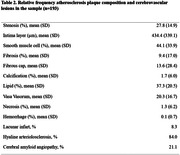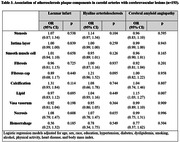# Atherosclerosis in carotid arteries associated with cerebrovascular lesions

**DOI:** 10.1002/alz.086932

**Published:** 2025-01-03

**Authors:** Beatriz Carvalho Pontes, Renata Elaine Paraizo Leite, Daniela Souza Farias‐Itao, Maria Eduarda Barbosa, Maria Eduarda Braga, Maristella Yahagi‐Estevam, Vitor Ribeiro Paes, Alberto Fernando Oliveira Justo, Carlos Augusto Pasqualucci, Lea T. Grinberg, Ricardo Nitrini, Wilson Jacob‐Filho, Claudia Kimie Suemoto

**Affiliations:** ^1^ University of São Paulo Medical School, São Paulo Brazil; ^2^ Physiopathology in Aging Laboratory (LIM‐22), Department of Pathology, University of São Paulo Medical School, São Paulo Brazil; ^3^ University of Sao Paulo, Sao Paulo Brazil; ^4^ University of São Paulo Medical School, São Paulo, Selecione Brazil; ^5^ University of São Paulo Medical School, São Paulo, Brazil, Sao Paulo Brazil; ^6^ Physiopathology in Aging Laboratory (LIM‐22), Department of Internal Medicine, University of Sao Paulo Medical School, São Paulo, São Paulo Brazil; ^7^ University of São Paulo Medical School, São Paulo, São Paulo Brazil; ^8^ Biobank for Aging Studies of the University of São Paulo Medical School, Brazil, São Paulo Brazil; ^9^ Department of Pathology, University of Sao Paulo Medical School, São Paulo, São Paulo Brazil; ^10^ Weill Institute for Neurosciences, University of California San Francisco, San Francisco, CA USA; ^11^ Biobank for aging studies of the University of São Paulo, São Paulo Brazil; ^12^ Division of Geriatrics, Department of Internal Medicine, University of Sao Paulo Medical School, São Paulo, São Paulo Brazil; ^13^ Division of Geriatrics, University of São Paulo Medical School, São Paulo, São Paulo Brazil

## Abstract

**Background:**

The atherosclerotic plaque in carotid arteries has been associated with dementia. Clinic radiological studies in older adults suggest that the composition of atherosclerotic plaque in the carotid artery can predict vascular dementia (VD) or mixed dementia. The proposed study aims to assess components of atherosclerotic plaques in the carotid arteries, particularly concerning cerebrovascular lesions using racially diverse autopsy samples.

**Method:**

We used data from the Biobank for Aging Studies at the University of São Paulo Medical School. We included participants aged 50 years or older at the time of death with a post‐mortem interval of less than 24 hours and a next of kin who had at least weekly contact with the deceased (n = 505). The plaque composition was evaluated using the Atherosclerotic Plaque Analyzer (APA) software. Cerebrovascular lesions included lacunar infarcts, hyaline arteriolosclerosis, and cerebral amyloid angiopathy evaluated microscopically in 13 samples areas. Logistic regression models adjusted for sociodemographic and clinical variables were used to investigate the associations between plaque composition and cerebrovascular deceased.

**Results:**

After the exclusions for missing data, 193 participants were included (mean age 79.4±10.0 years, 51.3% were women, and 61% were White) (Table 1). A higher percentage of lipid deposition in the plaque was associated with higher odds of cerebral amyloid angiopathy (OR = 1.01, 95% CI = 1.03; 1.27, p = 0.007). We found no association between the other plaque components and cerebrovascular diseases (Table 3).

**Conclusion:**

In an autopsy study, the deposition of lipids in the carotid atheroma plaque was associated with cerebral amyloid angiopathy. Further autopsy studies in larger samples are needed to confirm our findings.